# Combination Therapy of Acarbose and Cyclosporine a Ameliorates Imiquimod-Induced Psoriasis-Like Dermatitis in Mice

**DOI:** 10.3390/molecules25081822

**Published:** 2020-04-16

**Authors:** Hsin-Hua Chen, Chi-Chien Lin, Yu-Tang Tung, Ya-Hsuan Chao, Wen-Ching Huang, Po-Ying Lee

**Affiliations:** 1 Department of Medical Research, Taichung Veterans General Hospital, Taichung 407, Taiwan; shc5555@hotmail.com; 2 Institute of Biomedical Science and Rong Hsing Research Center for Translational Medicine, National Chung Hsing University, Taichung 402, Taiwan; lincc@dragon.nchu.edu.tw (C.-C.L.); demonsandy@gmail.com (Y.-H.C.); 3 Department of Medical Research, China Medical University Hospital, Taichung 404, Taiwan; 4 Graduate Institute of Metabolism and Obesity Sciences, Taipei Medical University, Taipei 110, Taiwan; f91625059@tmu.edu.tw; 5 Nutrition Research Center, Taipei Medical University Hospital, Taipei 110, Taiwan; 6 Cell Physiology and Molecular Image Research Center, Wan Fang Hospital, Taipei Medical University, Taipei 110, Taiwan; 7 Department of Exercise and Health Science, National Taipei University of Nursing and Health Sciences, Taipei 112, Taiwan; wenching@ntunhs.edu.tw; 8 Division of Plastic Surgery, Department of Surgery, Cathay General Hospital, Taipei 280, Taiwan

**Keywords:** acarbose, cyclosporine, psoriasis, imiquimod, combination therapy

## Abstract

Moderate to severe psoriasis, an immune-mediated inflammatory disease, adversely affects patients’ lives. Cyclosporin A (CsA), an effective immunomodulator, is used to treat psoriasis. CsA is ineffective at low doses and toxic at high doses. Acarbose (Acar), a common antidiabetic drug with anti-inflammatory and immunomodulatory effects, reduces imiquimod (IMQ)-induced psoriasis severity. Combinations of systemic drugs are generally more efficacious and safer than higher doses of single drugs. We observed that mice treated with a combination of Acar (250 mg/kg) and low-dose CsA (10 or 20 mg/kg) exhibited significantly milder IMQ-induced psoriasis-like dermatitis and smoother back skin than those treated with Acar (250 mg/kg), low-dose CsA (10 or 20 mg/kg), or IMQ alone. The combination therapy significantly reduced serum and skin levels of Th17-related cytokines (interleukin (IL)-17A, IL-22, and IL-23) and the Th1-related cytokine tumor necrosis factor-α (TNF-α) compared with Acar, low-dose CsA, and IMQ alone. Additionally, the combination therapy significantly reduced the percentages of IL-17- and IL-22-producing CD4^+^ T-cells (Th17 and Th22 cells, respectively) and increased that of Treg cells. Our data suggested that Acar and low-dose CsA in combination alleviates psoriatic skin lesions by inhibiting inflammation. The findings provide new insights into the effects of immunomodulatory drugs in psoriasis treatment.

## 1. Introduction

Psoriasis, a T-cell-mediated inflammatory immune disease, is characterized by a thickened epidermis resulting from hyperproliferative keratinocytes, parakeratosis, hyperkeratosis, and inflammatory leukocyte infiltration into the dermis and epidermis [1]. Psoriasis affects approximately 2% of the general population and 0.1%–0.3% of the Asian population [2,3,4,5], and it is associated with several comorbidities, such as cardiovascular disease, obesity, diabetes, hypertension, dyslipidemia, metabolic syndrome, nonalcoholic fatty liver disease, cancer, anxiety and depression, inflammatory bowel disease, and psoriatic arthritis [6].

The precise cellular and molecular mechanisms contributing to psoriasis are not completely understood; nevertheless, complex possible mechanisms involve interactions between inflammatory cytokines and infiltrating immune cells, including T-cells [7,8]. Tumor necrosis factor (TNF)-α, which is involved in the development of psoriasis, is overproduced by keratinocytes, and excess TNF-α increases Th17 cell generation [9]. In addition, Th17 cells and Th17-related cytokines, such as interleukin (IL)-17A, IL-22, and IL-23, are involved in the pathogenesis of psoriasis [10,11]. These cytokines are present in serum and skin lesions of patients with psoriasis and a mouse model of psoriasis-like dermatitis [12,13,14].

Cyclosporin A (CsA) is an effective immunomodulator. However, because of its long-term toxic effects, such as nephrotoxicity and hypertension, its use is restricted; moreover, the therapeutic effects of CsA disappear when treatment is stopped [15]. A combination therapy of CsA and other conventional systemic drugs can reduce the required drug dosage and side effects of each drug [16]. Acarbose (Acar) is a drug used to treat type 2 diabetes mellitus. If not used simultaneously with other antidiabetic drugs, Acar does not cause hypoglycemia [17]. Acar can reduce the levels of inflammatory markers in patients with diabetes [18,19,20]. Chen et al. [21] also reported that Acar can reduce the severity of imiquimod (IMQ)-induced psoriasis-like dermatitis through its anti-inflammatory and immunomodulatory effects. The aim of the present study was to investigate the therapeutic effects of Acar combined with low-dose CsA on IMQ-induced psoriasis-like dermatitis and to examine the molecular mechanisms underlying these effects.

## 2. Results

### 2.1. Effect of Single Doses of Acar and CsA on IMQ-Induced Psoriasis-Like Dermatitis in Mice

**Figure 1:**
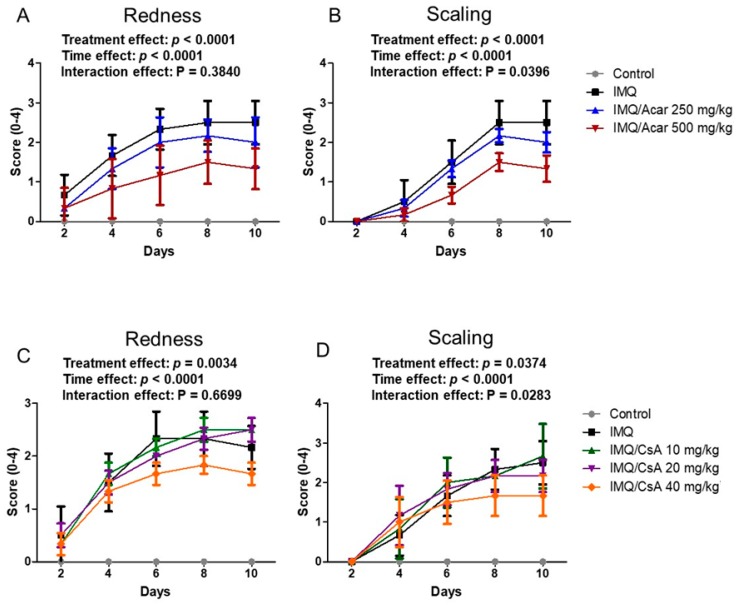
Effects of a single dose of Acarbose (Acar) (250 or 500 mg/kg) or Cyclosporin A (CsA) (10, 20, or 40 mg/kg) on skin lesions of mice with imiquimod (IMQ)-induced psoriasis-like dermatitis. The symptoms of redness (**A**,**C**) and scaling (**B**,**D**) were assessed. IMQ-treated mice and control mice received a daily dose of 62.5 mg of IMQ cream and control (vaseline) cream, respectively, on their shaved back skin for 9 days consecutively. Acar or CsA was administered to the mice orally once daily for 9 days consecutively by gavage in 200 μL of water. Experimental data are expressed as mean ± SD (*n* = 6). A mixed design two-way ANOVA was also applied to the supplementation effects within repeated time points.

To assess the therapeutic efficacy of Acar and CsA on IMQ-induced psoriasis-like dermatitis in mice, a single dose of Acar (250 or 500 mg/kg) or a single dose of CsA (10, 20, or 40 mg/kg) was orally administered to mice with dermatitis daily for 9 days. The severity of lesions was evaluated using an objective scoring system developed according to the Psoriasis Area and Severity Index (PASI); specifically, the degree of redness or scaling was assessed through a 5-point scale with scores ranging from 0 to 4 (Figure 1). IMQ cream and vaseline cream were applied to the shaved back skin of mice for 9 days consecutively. After the application of IMQ to the back skin of mice for 4 days, these areas began to exhibit redness and scaling (Figure 1). These symptoms of inflammation observed in mice treated with IMQ alone phenotypically resembled those of psoriasis. Acar (250 or 500 mg/kg) or CsA (10, 20, or 40 mg/kg) significantly ameliorated the symptoms of IMQ-induced psoriasis-like dermatitis in a dose-dependent manner (*p* < 0.05) (Figure 1). Accordingly, a high dose of Acar (500 mg/kg) or of CsA (40 mg/kg) markedly ameliorated redness and scaling. However, Acar (250 mg/kg) or low-dose CsA (10 or 20 mg/kg) did not have a significant inhibitory effect on redness and scaling in the mice. In addition, it showed significant difference in the time and supplementation (Acar or CsA) main effects with redness and scaling indexes (Figure 1). Therefore, Acar (250 mg/kg) and low-dose CsA (10 or 20 mg/kg), alone or in combination, were studied in subsequent experiments.

### 2.2. Effects of Combination of Acar and Low-Dose CsA on IMQ-Induced Psoriasis-Like Dermatitis in Mice

**Figure 2:**
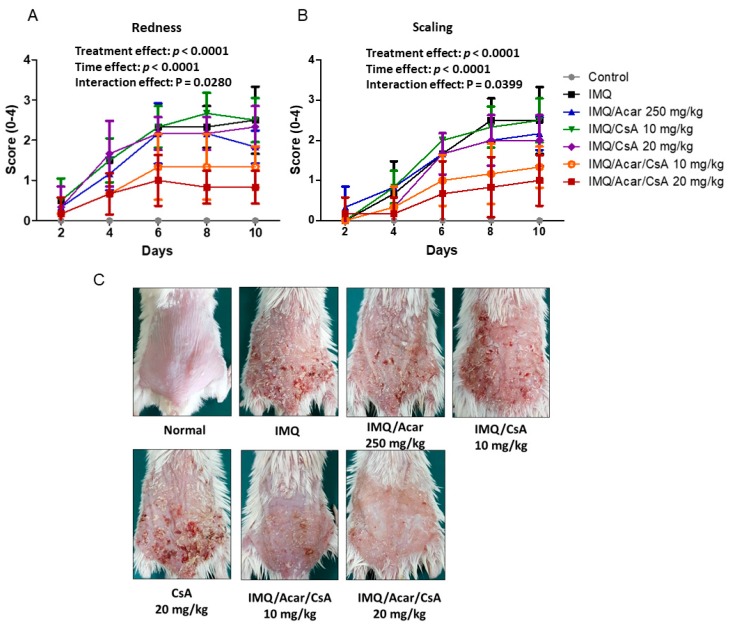
Effects of Acar (250 or 500 mg/kg) and/or low-dose CsA (10 or 20 mg/kg) on skin lesions of mice with IMQ-induced psoriasis-like dermatitis. Effects on symptoms of redness (**A**) and scaling (**B**); (**C**) representative images of the mice. time points. IMQ-induced mice and control mice received a daily dose of 62.5 mg of IMQ cream and control cream, respectively, on shaved back skin for 9 days consecutively. IMQ-induced mice were randomly assigned to one of the following six groups (n = 6): IMQ alone, Acar (250 mg/kg/day), CsA (10 mg/kg/day), CsA (20 mg/kg/day), Acar (250 mg/kg/day) + CsA (10 mg/kg/day), and Acar (250 mg/kg/day) + CsA (20 mg/kg/day). Acar was administered to the mice orally once a day for 9 days consecutively by gavage in 200 μL of water, either alone or in combination with CsA. Experimental data are expressed as mean ± SD (*n* = 6). A mixed design two-way ANOVA was also applied to the supplementation effects within repeated.

To examine the effects of the combination of Acar and low-dose CsA on IMQ-induced psoriasis-like dermatitis in mice, mice treated with Acar (250 mg/kg) and/or low-dose CsA (10 or 20 mg/kg) once a day for 9 days and the representative clinical features (redness and scaling) of IMQ-induced psoriasis-like dermatitis were assessed (Figure 2). The clinical features in the mice treated with the combination of Acar (250 mg/kg) and low-dose CsA (10 or 20 mg/kg) were notably milder than those in the mice treated with IMQ alone (*p* < 0.05); the back skin of the mice treated with Acar (250 mg/kg) and low-dose CsA (10 or 20 mg/kg) was smoother (Figure 2). However, no significant difference was observed between the mice treated with Acar alone (250 mg/kg), low-dose CsA alone (10 or 20 mg/kg), and IMQ alone. In addition, it showed significant difference in the time and supplementation (Acar, CsA, and Acar/CsA) main effects with redness and scaling indexes.

### 2.3. Effect of Combination of Acar and Low-Dose CsA on Histological Changes in the Skin of Mice with IMQ-Induced Psoriasis-Like Dermatitis

**Figure 3:**
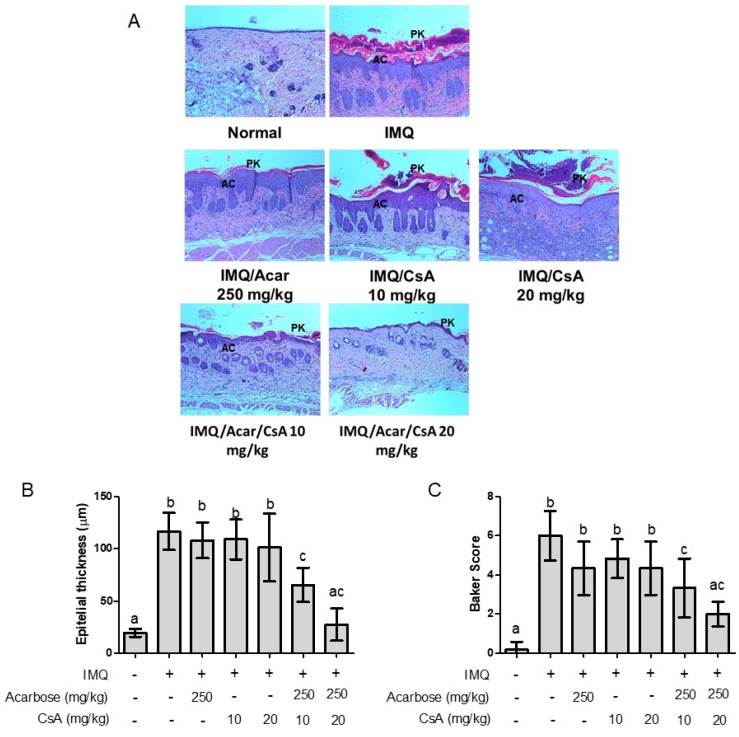
Effects of Acar (250 or 500 mg/kg) and/or low-dose CsA (10 or 20 mg/kg) on (**A**) histological features of skin lesions, (**B**) epithelial thickness, and (**C**) baker score of mice with IMQ-induced psoriasis-like dermatitis. IMQ-treated mice and control mice received a daily dose of 62.5 mg of IMQ cream and control cream, respectively, on shaved back skin for 9 days consecutively. IMQ-treated mice were randomly assigned to the following six groups (*n* = 6): IMQ alone, Acar (250 mg/kg/day), CsA (10 mg/kg/day), CsA (20 mg/kg/day), Acar (250 mg/kg/day) + CsA (10 mg/kg/day), and Acar (250 mg/kg/day) + CsA (20 mg/kg/day). Acar was administered to the mice orally once a day for 9 days consecutively by gavage in 200 μL of water, either alone or in combination with CsA. Experimental data are expressed as mean ± SD (*n* = 6). Columns with different superscript letters (a, b, c, ac) are significantly different at *p* < 0.05.

To further assess the effect of the combination of Acar and low-dose CsA on the severity of IMQ-induced psoriasis-like dermatitis, the degree of skin inflammation was determined through histopathological evaluation. The histological features of IMQ-induced psoriasis-like dermatitis in dorsal skin included hyperkeratosis, parakeratosis, modest acanthosis, elongated rete ridges, and modest dermal inflammation (Figure 3). However, the combination therapy of Acar (250 mg/kg) and low-dose CsA (10 or 20 mg/kg) ameliorated these symptoms more effectively than did treatment with Acar alone or low-dose CsA alone. In addition, the combination of Acar (250 mg/kg) and low-dose CsA (10 or 20 mg/kg) had a significantly lower epithelial thickness and baker score than those in the mice treated with Acar alone (250 mg/kg), low-dose CsA alone (10 or 20 mg/kg), and IMQ alone. (*p* < 0.05).

### 2.4. Effect of Combination of Acar and Low-Dose CsA on Levels of Inflammatory Cytokines in Serum and Skin of Mice with IMQ-Induced Psoriasis-Like Dermatitis

**Figure 4:**
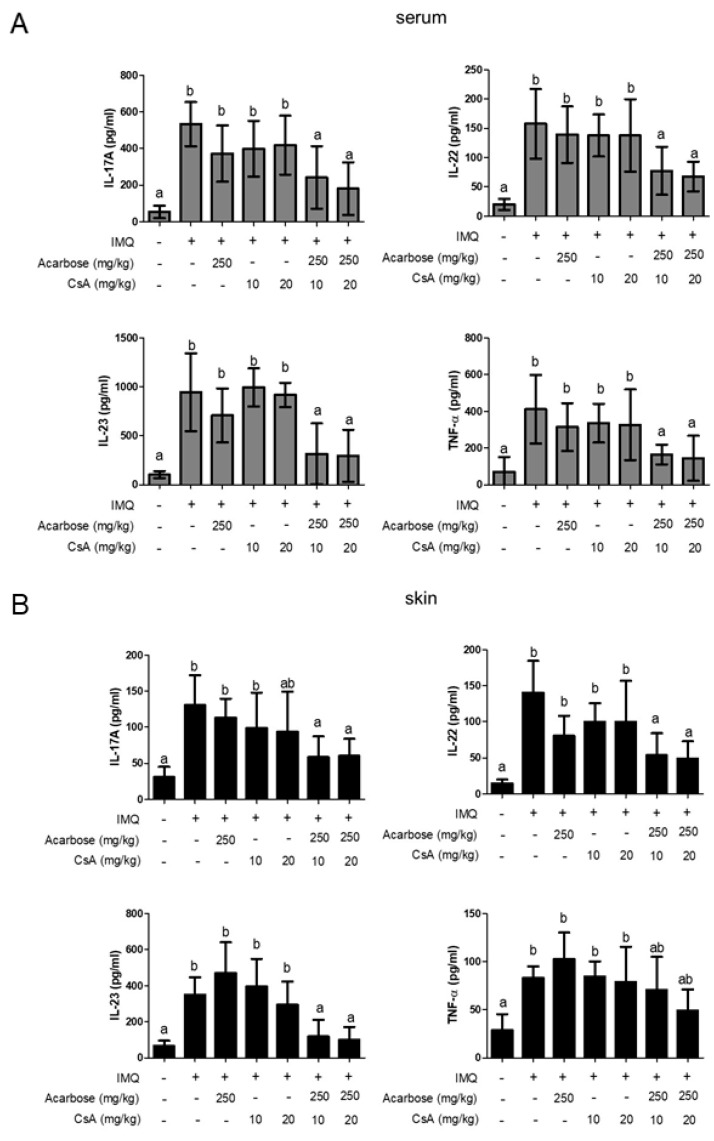
Effect of Acar (250 mg/kg) and/or low-dose CsA (10 or 20 mg/kg) on serum and skin cytokines in mice with IMQ-induced psoriasis-like dermatitis on a daily basis for 9 days. The levels of interleukin (IL)-17A, IL-22, IL-23, and tumor necrosis factor (TNF)-α in the serum (**A**) and skin (**B**) were measured using ELISA. IMQ-induced mice were randomly assigned to 6 groups (*n* = 6): IMQ alone, Acar (250 mg/kg/day), CsA (10 mg/kg/day), CsA (20 mg/kg/day), Acar (250 mg/kg/day) + CsA (10 mg/kg/day), and Acar (250 mg/kg/day) + CsA (20 mg/kg/day). The mice were subjected to an intervention with either Acar or CsA alone or in combination per day for 9 consecutive days orally. Experimental data are expressed as the mean ± SD (*n* = 6) and data analysis was performed with Mann–Whitney U test. Columns with different superscript letters (a, b, ab) are significantly different at *p* < 0.05.

To investigate the therapeutic efficacy of the combination of Acar and low-dose CsA on immune responses, the levels of inflammatory cytokines in the serum and skin of the mice treated with drugs alone or in combination were assessed (Figure 4). The mice treated with the combination of low-dose (10 or 20 mg/kg) CsA and Acar (250 mg/kg) exhibited significantly lower serum and skin levels of Th17-related cytokines, including IL-17A, IL-22, and IL-23, than did those treated with Acar alone or low-dose CsA alone (*p* < 0.05). In addition, the combination of low-dose (10 or 20 mg/kg) CsA and Acar (250 mg/kg) significantly reduced the levels of the Th1-related cytokine TNF-α in the serum (*p* < 0.05). However, skin TNF-α level in the mice treated with the combination of low-dose (10 or 20 mg/kg) CsA and Acar (250 mg/kg) did not differ significantly from that in the mice treated with IMQ alone.

### 2.5. Effect of Combination of Acar and Low-Dose CsA on the Percentages of CD4^+^ IL-17A^+^ T-cells, CD4^+^ IL-22^+^ T-Cells, and Treg Cells in Mice with IMQ-Induced Psoriasis-Like Dermatitis

**Figure 5:**
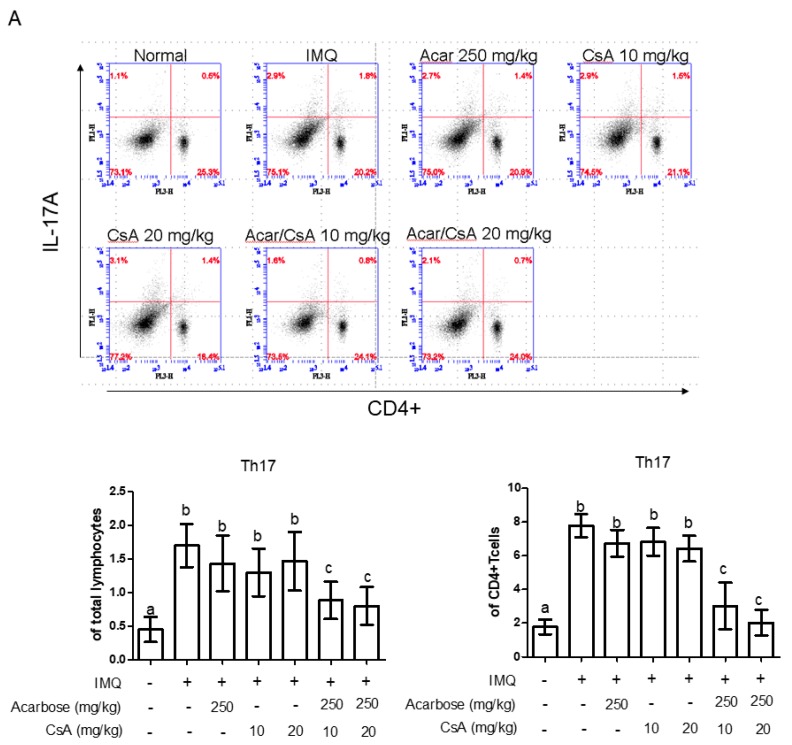

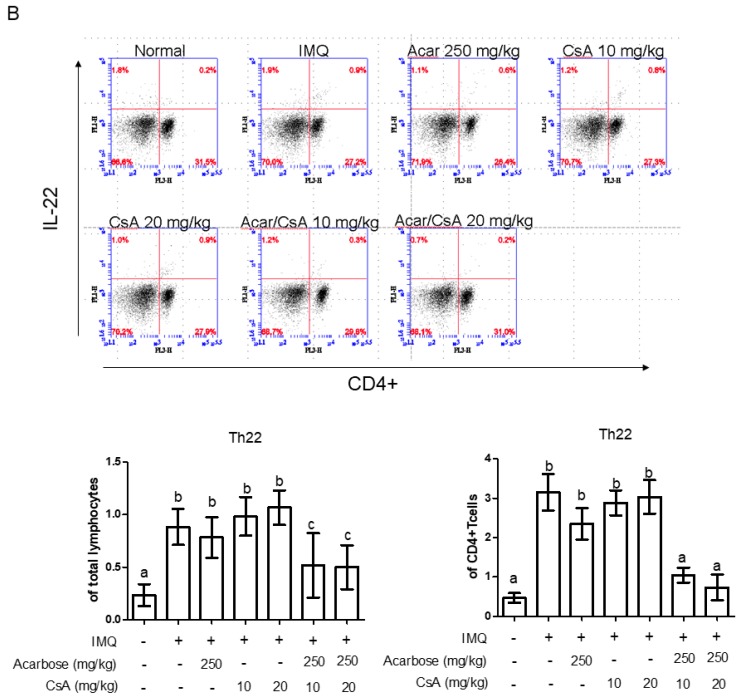

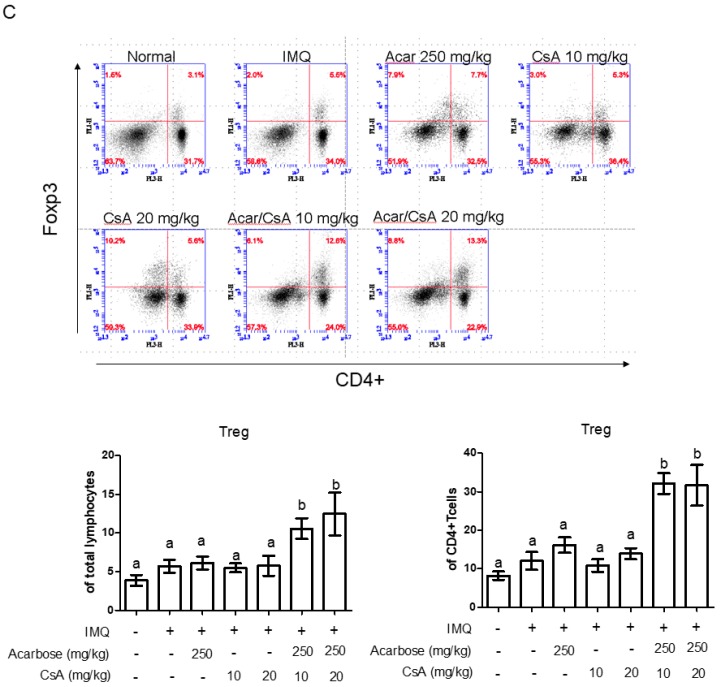
Effects of Acar (250 or 500 mg/kg) and/or low-dose CsA (10 or 20 mg/kg) on IL-17A^+^ T-cells (**A**), CD4^+^ IL-22^+^ T-cells (**B**), and Treg cells (**C**) in mice with IMQ-induced psoriasis-like dermatitis. IMQ-induced mice were randomly assigned to the following six groups (*n* = 6): IMQ alone, Acar (250 mg/kg/day), CsA (10 mg/kg/day), CsA (20 mg/kg/day), Acar (250 mg/kg/day) + CsA (10 mg/kg/day), and Acar (250 mg/kg/day) + CsA (20 mg/kg/day). Acar was administered to the mice orally once a day for 9 consecutive days by gavage in 200 μL of water, either alone or in combination with CsA. Experimental data are expressed as mean ± SD (*n* = 6), and data analysis was performed using the Mann–Whitney U test. Columns with different superscript letters (a, b, c) are significantly different at *p* < 0.05.

In psoriasis, CD4^+^ cells are the main source of IL-17 and IL-22 [22,23]. Therefore, we evaluated the effects of Acar combined with low-dose CsA on CD4^+^-T-cell-induced IL-17 and IL-22 production in mouse spleens after 9 days of IMQ treatment. IMQ-treated mice exhibited higher percentages of splenic CD4^+^ IL-17A^+^ T-cells and CD4^+^ IL-22^+^ T-cells than did the normal control mice (Figure 5A,B). However, the mice treated with the combination of low-dose CsA (10 or 20 mg/kg) and Acar (250 mg/kg) exhibited a significantly lower percentage of IL-17-producing CD4^+^ T-cells (Figure 5A) and IL-22-producing CD4^+^ T-cells (Figure 5B) than did those treated with Acar alone or low-dose CsA alone. Furthermore, no significant difference in the percentage of CD4^+^ IL-17A^+^ T-cells or CD4^+^ IL-22^+^ T-cells was observed between the mice treated with Acar alone, low-dose CsA alone, and IMQ alone. We also examined whether the combination therapy could induce the proliferation of CD4^+^ Foxp3^+^ Treg cells in the spleen. The percentages of CD4^+^ Foxp3^+^ T-cells were significantly higher in the mice treated with the combination therapy than in mice treated with IMQ alone (Figure 5C).

## 3. Discussion

Currently, the main treatments for psoriasis include psoralen–UVA photochemotherapy, topical therapies (steroid, retinoids, immunosuppressive agents, and vitamin D3 derivatives), and biological therapies [24,25,26,27]. However, most therapies have varying degrees of side effects and require long-term administration. The synergistic effects of various drug combinations have attracted considerable attention in the field of biomedicine; therefore, a combination drug therapy is becoming an increasingly attractive treatment option [28]. CsA is a first-line immunosuppressant used for treating autoimmune diseases. However, nephrotoxicity and hypertension resulting from high doses and long-term use considerably limit the use of CsA. Our previous study [21] found that Acar could significantly ameliorate the symptoms of IMQ-induced psoriasis-like inflammation in mice. Acarbose, a pseudotetrasaccharide, is poorly absorbed into the blood from the gut, the anti-inflammatory activity might be through its interaction with gut immune system. Although the present study did not investigate the influence of CsA and Acar on gut immune system, our previous study had shown that Acar could induce CD4^+^ IL10^+^ type 1 regulatory T-cells in intestinal lymphocytes in IMQ-treated mice [21]. However, our previous study also revealed that acarbose did not directly influence the capability of intestinal lamina propria dendritic cells from naïve BALB/c mice to induce CD4^+^ IL-10^+^ T-cell development in vitro [21]. Interestingly, Acar was known to have effect on the immune system. Therefore, the co-administration of CsA and Acar may enhance the immunosuppressive effects. Mice were given CsA subcutaneously for 6 wk at a dose of 12.5, 50 or 200 mg/kg/d, and no abnormalities were observed in mice given CsA 12.5 mg/kg/d [29]. Combination drug therapy effectively reduces drug toxicity and improves drug efficacy [28]. Therefore, in the present study, we investigated the therapeutic effects of Acar (250 mg/kg) combined with low-dose CsA (10 or 20 mg/kg) on IMQ-induced psoriasis-like dermatitis.

An IMQ-induced psoriasis mouse model in a previous study closely resembled human psoriasis in terms of not only phenotypic symptoms but also histological features, including erythema, skin thickening, scaling, epidermal alterations (acanthosis and parakeratosis), and neoangiogenesis; the presence of inflammatory cells comprising T-cells, neutrophils, and dendritic cells; and the presence of vascular proliferation [30]. According to our results, IMQ-induced psoriasis-like dermatitis was successfully established in the IMQ-treated mice. We also found that treatment with a combination of Acar (250 mg/kg) and low-dose CsA (10 or 20 mg/kg) significantly inhibited the development of IMQ-induced psoriasis-like dermatitis in mice compared with treatment with Acar alone or low-dose CsA alone. Kim et al. [15] showed that combined treatment with Glu (300 mg/kg) and low-dose CsA (10 or 20 mg/kg) strongly ameliorated the development of psoriasis-like skin lesions. Therefore, combination therapy with CsA may be an effective choice for improving drug efficacy and reducing drug toxicity.

Destruction of Treg cell function is involved in the development of various inflammatory skin diseases, including psoriasis [31]. As revealed by our results, treatment with a combination of Acar (250 mg/kg) and low-dose CsA (10 or 20 mg/kg) significantly increased the percentage of Treg cells compared with treatment with Acar alone or low-dose CsA alone. Thus, the combination therapy could increase the number of Treg cells available for suppressing inflammatory factors, including Th17-related cytokines. We also found that the combination treatment significantly increased the percentages of IL-17-producing CD4^+^ T-cells and IL-22-producing CD4^+^ T-cells compared with treatment with Acar alone or low-dose CsA alone. In addition, Th17 responses are involved in IMQ-induced psoriasis-like lesions, and the combination of Acar and low-dose CsA could significantly modulate the serum and skin expression levels of Th17 cytokines (IL-17A, IL-22, and IL-23) compared with Acar alone or low-dose CsA alone. The development of psoriasis is associated with the IL-23–IL-17 axis [30] and is mediated by Th17 cells [32]. Th17 is a group of IL-17-secreting CD4^+^ T-cells observed in inflammatory or autoimmune diseases [1]. IL-23 is mainly derived from activated macrophages and dendritic cells and is a key cytokine that promotes Th17 cell differentiation and development [33]. Blauvelt [34] showed that patients with psoriasis exhibited markedly high percentages of Th17 cells and Th17 cytokine levels in serum. Furthermore, TNF-α is involved in the development of psoriasis and hyperproliferation of keratinocytes and enhances Th17 cell generation [9]. These results demonstrated that the combination therapy of Acar and low-dose CsA could inhibit the development of IMQ-induced psoriasis-like dermatitis in mice and reduce the levels of Th1-related cytokines (TNF-α) and Th17-related cytokines (IL-17A, IL-22, and IL-23) in serum and skin compared with treatment with Acar alone or low-dose CsA alone. Chen et al. [21] showed that Acar treatment in mice markedly suppressed IL-17A and IL-22 production in inflamed skin and reduced the percentages of Th17 cells and IL-17- and IL-22-producing γδ Τ cells in the spleen. The combination therapy of Glu (300 mg/kg) and low-dose (10 or 20 mg/kg) CsA could reduce the levels of TNF-α, IL-17A, IL-22, and IL-23. Therefore, CsA in combination with another drug may be an effective choice to improve drug efficacy. In the future, we should need to investigate the pharmacokinetics and toxicity of the combination therapy with CsA and Acar.

However, some limitations should be addressed. First, although we assessed the efficacy of the CsA combined with Acar by scoring of skin redness and scaling, we did not investigate their effect on pruritus, which is also an important psoriasis related symptom. Further studies are warranted to investigate whether or not CsA combined with Acar can provide additional effect on pruritus using other murine models, such as dinitrofluorobenzene-induced atopic dermatitis model [35]. Second, further mechanistic experiments using other in vivo as well as in vitro models of skin inflammation and psoriasis-specific dermatitis are warranted. CsA forms a complex with cyclophilin. The main immunomodulatory effect of CsA-cyclophilin complexes is via blocking T-cell activation by calcineurin inhibition with downregulation of store-operated (or capacitative) Ca^2+^ entry (SOCE) and translocation of the transcription factor nuclear factor of activated T-cells (NFAT) from the cytoplasm to the nucleus. [35,36,37]. Karvonen et al. [38] found a downregulated capacitative calcium influx and defective calcium-mediated cell signaling in cultured psoriatic keratinocytes. Given that the CsA binding proteins cyclophilin A is also expressed by keratinocytes and nonimmune cells, Al-Daraji et al. [39] revealed that the calcineurin is functionally active in human keratinocytes inducing nuclear translocation of NFAT1 and suggested a possible therapeutic effect of CsA by inhibition of this pathway in epidermal keratinocyte. Taken together, further experiments using cultured psoriatic keratinocytes to assess the possible synergistic effect of CsA and Acar on the calcium-clacineurin-NFAT signaling pathway are also warranted. Given the complex interplay between epidermal barrier alterations and immune dysregulations in psoriasis, further in vitro studies using various 2D and 3D models may help to investigate the therapeutic effect of CsA combined with Acar on the crosstalk between keratinocytes and immune cells [40]. Third, given that acarbose is poorly absorbed, the anti-inflammatory activity might be through its interaction with gut immune system at different levels such as intestinal microbiota [41] or regulatory networks of glucose metabolism and inflammation [42]. Further experiments at the two levels in gut are also warranted to elucidate the mechanisms of the therapeutic effect of CsA combined with Acar.

## 4. Materials and Methods

### 4.1. Animal Treatment Procedures

Male BALB/c mice (aged 6 weeks) were provided by the National Laboratory Animal Center (Taipei, Taiwan). The animals were housed in an animal housing facility at 22 ± 2 °C with 60%–70% relative humidity and a 12-h light/12-h dark cycle. The mice were maintained under specific pathogen-free conditions and fed a chow diet (Lab 5001, vendor info) and water ad libitum. They were adapted for 1 week before the initiation of experiments. All experiments were approved by the Institutional Animal Care and Use Committee (IACUC) of National Chung Hsing University, Taichung, Taiwan (IACUC no. 105-035).

In this study, the therapeutic effects of Acar (Sigma-Aldrich, St. Louis, MO) combined with low-dose CsA (Sigma-Aldrich, St. Louis, MO) on IMQ-induced psoriasis-like dermatitis in mice were evaluated. At the age of 7 weeks, 54 mice were randomly assigned to control mice (*n* = 6) and IMQ-induced mice (*n* = 48). IMQ-induced mice and control mice received a daily dose of 62.5 mg of IMQ cream (5%; Aldara; 3M Pharmaceuticals, St. Paul, MN, USA) and control cream (vaseline cream; Unilever, Greenwich, CT, USA), respectively, on the shaved back skin for 9 consecutive days. IMQ-induced mice were randomly assigned to 8 groups (*n* = 6): IMQ alone, Acar (250 mg/kg/day), Acar (500 mg/kg/day), CsA (10 mg/kg/day), CsA (20 mg/kg/day), CsA (40 mg/kg/day), Acar (250 mg/kg/day) + CsA (10 mg/kg/day), and Acar (250 mg/kg/day) + CsA (20 mg/kg/day). Acar was administered to the mice orally once a day for 9 consecutive days by gavage in 200 μL of water, either alone or in combination with CsA.

### 4.2. Scoring of Skin Inflammation Severity

To assess the severity of back skin inflammation, an objective scoring system was developed according to the Clinical PASI; all back skin areas were considered in the assessment, except for the affected skin areas. Redness and scaling were scored on days 2, 4, 6, 8, and 10 by an independent investigator blinded to the treatment groups; a scale from 0 to 4 (0 = none; 1 = slight; 2 = moderate; 3 = marked; 4 = very marked) was used for scoring.

### 4.3. Pathological Histology

The mice with IMQ-induced dermatitis-like psoriasis treated with Acar, CsA, Acar + CsA, or vehicle were euthanized, and their back skin was removed, fixed in 4% paraformaldehyde solution, and formally embedded in paraffin. The paraffin-embedded 6-μm-thick sections were examined using hematoxylin and eosin staining through light microscopy. The epidermal thickness is measured using ZAKImage 2016 software (zak microscope, Taichung, Taiwan). The Baker scoring criteria was followed the previous study [43] to analyze the histopathological changes.

### 4.4. Assay of Cytokines Production

For measuring cytokine levels in serum, the collected serum was stored at −80 °C until analysis. Subsequently, skin samples were homogenized using a Bullet Blender^TM^ (Next Advance, Averill Park, NY, USA) at 4 °C, and the supernatants were stored at −80 °C. Serum and skin IL-17A, IL-22, IL-23, and TNF-α levels were measured using specific enzyme-linked immunosorbent assay kits for IL-17A (R&D system, Minneapolis, MN, USA), IL-22 (eBioscience, San Diego, CA, USA), IL-23 (eBioscience, San Diego, CA, USA), and TNF-α (eBioscience, San Diego, CA, USA). The protocols followed were provided by the manufacturer.

### 4.5. Intracellular Cytokine Staining and Flow Cytometry

At the end of the experiments, the excised spleens of the euthanized mice were minced using a 200-mm mesh screen to obtain single-cell suspensions. For intracellular detection of cytokines, 2 × 10^5^ cells/well were stained using a phycoerythrin-conjugated antimouse CD4 (GK1.5, Biolegend, San Diego, CA, USA) antibody, followed by fixation. Intracellular staining was performed using the Cytofix/Cytoperm Plus Kit (BD Biosciences, San Diego, CA, USA) according to the manufacturer’s instructions. The cells were then stained intracellularly with the fluorescein-isothiocyanate-conjugated mAbs specific to mouse IL-17A (TC11-18H10.1) and IL-22 (Poly5164) and the Alexa Fluor^®^ 488-conjugated mAb specific to mouse Foxp3 (150D). The stained cells were detected on a BD Accuri™ C5 cytometer CAT. NO. 657,214. (BD Biosciences, San Jose, CA, USA) and analyzed for expression levels of phenotypic markers using BD Accuri™ C6 software version 1.0264.21 (BDBioscience, San Jose, CA, USA).

### 4.6. Statistical Analysis

Experimental data are expressed as mean ± SD (*n* = 6). Data analysis was performed through the Mann–Whitney *U* test using GraphPad Prism version 5.0 (GraphPad Software; San Diego, CA, USA). A *p*-value of <0.05 was considered statistically significant. A mixed design two-way ANOVA (supplementation × time) was also applied to the supplementation effects within repeated time points.

## 5. Conclusions

The results of the present study demonstrate that Acar combined with low-dose CsA exhibits remarkable potential in efficaciously ameliorating the symptoms of and attenuating the processes underlying psoriasis-like dermatitis in mice. The combination therapy attenuates IMQ-induced psoriasis-like inflammation by downregulating the levels of IL-17A, IL-22, IL-23, and TNF-α in the serum and skin and upregulating Treg cells compared with Acar alone or low-dose CsA alone. The results suggest that the synergistic effect of Acar and low-dose CsA in ameliorating IMQ-induced psoriasis-like dermatitis warrants special attention, and combination therapy can provide an alternative immunomodulatory approach for treating T-cell-mediated autoimmune diseases, including psoriasis. Further in vivo studies and in vitro studies, including cultured psoriatic keratinocyte models, are needed to assess its effects on pruritus and the mechanisms of its anti-inflammation effects, respectively.
